# Inhibitory Effect of *Asplenium incisum* on Bacterial Growth, Inflammation, and Osteoclastogenesis

**DOI:** 10.3390/medicina57070641

**Published:** 2021-06-22

**Authors:** Seong-Hee Moon, Ju-Lee Son, Seong-Jin Shin, Seung-Han Oh, Seong-Hwan Kim, Ji-Myung Bae

**Affiliations:** 1 Department of Dental Biomaterials, Institute of Biomaterials & Implant, College of Dentistry, Wonkwang University, 460 Iksan-daero, Iksan 54538, Korea; shmoon06@gmail.com (S.-H.M.); shoh@wku.ac.kr (S.-H.O.); 2 Department of Dental Hygiene, Wonkwang Health Science University, 514 Iksan-daero, Iksan 54538, Korea; julee890716@naver.com; 3 Department of Dental Biomaterials, College of Dentistry, Wonkwang University, 460 Iksan-daero, Iksan 54538, Korea; ko2742@naver.com; 4 Innovative Target Research Center, Bio & Drug Discovery Division, Korea Research Institute of Chemical Technology, 141 Gajeong-ro, Yuseong-gu, Daejeon 34114, Korea; hwan@krict.re.kr

**Keywords:** antibacterial, *Asplenium incisum*, inflammation, periodontitis, osteoclast differentiation

## Abstract

*Background and Objectives:Asplenium incisum,* a natural plant, is known to possess numerous pharmacological and biochemical properties. However, the inhibitory effect of *A. incisum* against *Porphyromonas gingivalis* and other factors related to periodontal disease have not yet been demonstrated. This study aimed to investigate the potential of *A. incisum* extract as a phytotherapeutic candidate for improving periodontal diseases by assessing its antibacterial, anti-inflammatory, and anti-osteoclastogenic activities. *Materials and Methods*: The inhibition of proliferation of *P. gingivalis* by *A. incisum* and the sustainability of its antibacterial activity were evaluated in this study. The production of inflammatory cytokines (tumor necrosis factor-α (TNF-α) and interleukin-6 (IL-6)) and nitric oxide (NO) from lipopolysaccharide-stimulated RAW 264.7 cells was assessed using an enzyme-linked immunosorbent assay. To identify the anti-osteoclastogenic activity, tartrate-resistant acid phosphatase (TRAP) staining and TRAP activity analyses were performed on bone marrow macrophages. *Results*: The proliferation of *P. gingivalis* was significantly inhibited by *A. incisum* (*p* < 0.001), and the antibacterial activity was sustained for up to 3 days. *A. incisum* showed anti-inflammatory activities by significantly decreasing the release of TNF-α, IL-6 (*p* < 0.05), and NO (*p* < 0.01). In addition, *A. incisum* significantly suppressed TRAP-positive cells and TRAP activity (at 30 μg/mL, *p* < 0.01) without causing any cytotoxicity (*p* > 0.05). *Conclusions:* *A. incisum* showed antibacterial, anti-inflammatory, and anti-osteoclastogenic activities, suggesting it has strong therapeutic potential against periodontal diseases.

## 1. Introduction

Periodontitis, which is a chronic inflammatory disease caused by periodontal pathogens, is one of the most prevalent diseases worldwide [1]. Well-known periodontal pathogens causing periodontal diseases include approximately 700 bacteria and, among them, *Porphyromonas gingivalis* is associated with periodontal diseases that cause alveolar bone loss [2]. Periodontitis is a multifactorial disease that occurs because of exposure to several cofactors, including bacterial and viral infections, inflammation, and genetic factors, as well as health behaviors and various social factors [3]. The pathological features of periodontitis include bone resorption owing to high levels of inflammatory cell infiltration and the breakdown of tooth-supporting tissue [4]. It is principally mediated by the production of proinflammatory cytokines (tumor necrosis factor (TNF-α), interleukin (IL)-6, and IL-1β), as well as the production of nitric oxide (NO) [5]. Therefore, the control of related pathogens, which would subsequently enable us to tune the inflammatory response and prevent further destruction, is crucial to the prevention of periodontitis, while treatments should aid in regenerating the periodontal tissue [6]. The initial treatment for periodontitis involves mechanical techniques, such as root planning [7]. However, in deep periodontitis pockets or furcation areas, complete elimination of bacteria might not be possible and, hence, additional antibiotic application may be advantageous [8]. Because this treatment relies primarily on antibiotics, it can cause many complications, including drug resistance, indigestion, and oral symptoms, such as swelling of the lips and tongue [9]. This has led to an increasing interest in phytotherapy using new natural therapeutic agents. Accordingly, the antibacterial activities of *Stemodia maritima* L. extract [10], propolis [11], and green tea extract [12], as well as the anti-inflammatory effect of bromelain [13], have been widely researched.

*Asplenium incisum* is a small perennial fern with short rhizomes belonging to the Aspleniaceae family [14]. The whole plant of *A. incisum* has shown various effects, such as detoxification, and the raw juice is used for medicinal purposes [15]. Wild plant extracts, which have few side-effects and show sustained therapeutic effects, are widely used in phytotherapy [16]. *A. incisum* has been reported to show antibacterial activity against bacteria causing skin diseases, bronchodilatory effects, and various pharmacological properties, including effectiveness in treating hepatitis, tinnitus, and chronic manganese poisoning [15]. However, the effect of *A. incisum* on preventing or improving oral diseases, including periodontitis, has not yet been elucidated. Therefore, research is necessary to determine whether *A. incisum* is effective in inhibiting the factors related to the onset of periodontitis.

In this study, we aimed to investigate the inhibitory effects of *A. incisum* on bacterial proliferation, inflammation, and osteoclastogenesis. To determine the antibacterial activities of *A. incisum*, we evaluated the inhibition of proliferation of *P. gingivalis* and the sustainability of its antibacterial effects. The anti-inflammatory effects were analyzed by measuring the release of proinflammatory cytokines, such as TNF-α and IL-6, as well as that of NO. In addition, the anti-osteoclastogenic activity of *A. incisum* was examined using tartrate-resistant acid phosphatase (TRAP) staining and a TRAP activity assay.

## 2. Materials and Methods

### 2.1. A. incisum Extract

**Figure 1:**
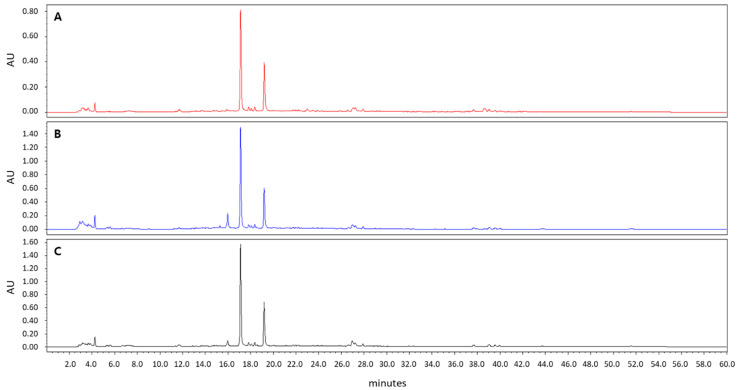
High-performance liquid chromatography chromatograms of *Asplenium incisum* according to the solvents used for extraction: (**A**) 100% methanol extract; (**B**) 75% ethanol extract; (**C**) 95% ethanol extract.

*A. incisum* was purchased from the Korea Plant Extract Bank at the Korea Research Institute of Bioscience and Biotechnology (KRIBB, Daejeon, Korea). After drying and powdering, *A. incisum* (79 g) was extracted with methanol (1 L) at room temperature by using an ultrasonic extractor (SDN-900H; SD-ULTRASONIC CO., LTD, Seoul, Korea). The methanol extract of *A. incisum* (8.73 g) was obtained by filtration under reduced pressure. A stock solution of 50 mg/mL was prepared by diluting the *A. incisum* extract in dimethyl sulfoxide, and it was stored at −20 °C until use. The methanol extract of *A. incisum* was subjected to high-performance liquid chromatography (HPLC) analysis using HPLC–PDA (Waters 2695 separation module; Waters, Milford, MA, USA) at 254 nm and was compared to the extracts with different solvents (75% and 95% ethanol) (Figure 1). A Phenomenex Luna C18 column (250 × 4.60 mm; 5 μm particle size) was used for HPLC, and the mobile phase was a mixture of water and 90% acetonitrile applied at a flow rate of 0.8 mL/min.

### 2.2. Antibacterial Assay and Sustainability of Antibacterial Activity

*P. gingivalis* (ATCC 33277) was provided by the Korean Collection of Type Cultures (KCTC) at the KRIBB. *P. gingivalis* was cultured in brain heart infusion medium supplemented with hemin (5 μg/mL), menadione (0.5 μg/mL), and sheep blood (5% *v/v*). *P. gingivalis* subcultures were incubated anaerobically at 37 °C for 3 days.

For the antibacterial assay, 90 μL of *P. gingivalis* (1.5 × 10^8^ colony-forming units (CFU)/mL) was prepared in a 96-well plate. Thereafter, 10 μL of the *A. incisum* extract was added to produce the following final concentrations: 0, 125, 250, and 500 μg/mL. After 3 days, the amount of inhibition of *P. gingivalis* proliferation was determined on the basis of the optical density (OD) measured using a microplate reader (SpectraMax 250; Molecular Devices Co., San Jose, CA, USA) at a wavelength of 600 nm.

For determining the sustainability of antibacterial activity, an experimental fluoride varnish (FV) was fabricated as a carrier for *A. incisum*. The experimental FV was formulated according to a previously reported method [17]. Briefly, the experimental FV was composed of 45 wt.% rosin, 50 wt.% ethanol, and 5 wt.% sodium fluoride. The groups were divided as follows: control, FV, and 50 mg/mL *A. incisum* extract in FV (FV + AI) groups. Polyethylene terephthalate film discs (5 mm diameter), sterilized using ethylene oxide gas, were used as controls. For the FV and FV + AI groups, 5 µL of each material was applied to the discs, and the discs (four discs/group) were placed in 35 mm diameter Petri dishes with 2 mL of distilled water. Subsequently, the materials on the film discs were eluted into distilled water in a 37 °C shaking water bath at 80 rpm for 3 days. To assess the sustainability of antibacterial activity, agar diffusion tests were performed by inoculating *P. gingivalis* (1.5 × 10^7^ CFU/mL) after removing the discs from the Petri dishes. After anaerobic incubation at 37 °C for 3 days, the average of the two perpendicular diameters of the inhibition zones of each disc was determined as the sustainability of antibacterial activity.

### 2.3. Anti-Inflammatory Activity by Measuring TNF-α, IL-6, and NO Levels

RAW 264.7 macrophages (American Type Culture Collection, Rockville, MD, USA) were incubated at 37 °C in a humidified 5% carbon dioxide (CO_2_) atmosphere in Dulbecco’s modified Eagle’s medium supplemented with 10% fetal bovine serum (FBS), penicillin (100 U/mL), and streptomycin (100 μg/mL). The cells (1 × 10^6^ cells) were cultured in a 60 mm dish for 24 h. Thereafter, the cells were treated with up to 30 μg/mL of the *A. incisum* extract for 2 h and incubated for 24 h with 1 μg/mL lipopolysaccharide (LPS; L4130; Sigma-Aldrich, St Louis, MO, USA).

To assess the secretion of TNF-α and IL-6, supernatants were collected from the cells and measured using enzyme-linked immunosorbent assay (ELISA) kits (R&D Systems, Minneapolis, MN, USA). In addition, NO levels in the cell culture medium were measured using the Griess reaction protocol [18]. Briefly, 100 μL of the supernatant was mixed with 100 μL Griess reagent (1% *w/v* sulfanilamide in 5% *v/v* phosphoric acid and 0.1% *w/v N*-(1-naphthyl) ethylenediamine dihydrochloride) and left to react for 10 min at room temperature. The OD was measured at 540 nm by using an ELISA reader (VersaMax; Molecular Devices Co.). Fresh culture medium was used to obtain blank readings. Nitrite levels in the samples were calculated using a standard sodium nitrite curve.

### 2.4. Anti-Osteoclastogenesis Assay Using TRAP Staining and TRAP Activity

Bone marrow cells (BMCs) were isolated from 5 week old male mice of the imprinting control region strain (Damool Science, Daejeon, Korea) according to the recommendations of the Standard Protocol for Animal Study of Korea Research Institute of Chemical Technology (KRICT). The protocol (ID No. 7D-M1 and 04/24/2014) was approved by the Institutional Animal Care and Use Committee of KRICT. BMCs were obtained by flushing the tibias and femurs of mice and were cultured with minimum essential medium Eagle’s alpha modification (α-MEM) containing 100 U/mL penicillin and 100 µg/mL streptomycin. Bone marrow macrophages (BMMs) were obtained by plating BMCs on a cell culture dish containing α-MEM supplemented with 10% FBS and macrophage colony-stimulating factor (M-CSF; 10 ng/mL) in a humidified 5% CO_2_ atmosphere at 37 °C for 24 h. Nonadherent cells were seeded on a Petri dish and cultured with M-CSF (30 ng/mL) for 3 days, and the adherent cells were used as BMMs [19]. To differentiate BMMs into osteoclasts, BMMs (1 × 10^4^ cells/well in a 96-well plate) were cultured for 4 days in a complete medium containing 30 ng/mL M-CSF, 10 ng/mL receptor activator of nuclear factor-κB ligand (RANKL), and 3, 10, and 30 μg/mL of the *A. incisum* extract. Thus, osteoclasts that were TRAP-positive multinucleated cells were obtained from BMMs.

For TRAP staining, the differentiated cells were fixed in 3.7% formaldehyde for 5 min, permeabilized with 0.1% Triton X-100 for 5 min, and stained with TRAP staining solution (Leukocyte Acid Phosphatase Kit 387-A; Sigma-Aldrich). The cells were then rinsed in distilled water, air-dried, and observed under a microscope.

TRAP activity was measured using the TRAP buffer (100 mM sodium citrate (pH 5.0) and 50 mM sodium tartrate) mixed with 3 mM *p*-nitrophenyl phosphate (Sigma-Aldrich). As described previously [20], 100 μL of the TRAP buffer was reacted with the permeabilized cells at 37 °C for 1 h. The reaction mixtures (50 μL) were immediately transferred into new plates containing 0.1 N sodium hydroxide (50 μL) in order to measure the OD at 405 nm by using a Wallac EnVision microplate reader (PerkinElmer, Waltham, MA, USA).

### 2.5. Cytotoxicity Test

BMMs (1 × 10^4^ cells/well in a 96-well plate) were cultured with M-CSF (30 ng/mL). After 24 h, the cells were incubated with each concentration of the *A. incisum* extract (3, 10, and 30 μg/mL) for 3 days. Cell viability was evaluated using the Cell Counting Kit-8 (Dojindo Molecular Technologies, Rockville, ML, USA). The OD was measured at 450 nm using a microplate reader (VersaMax; Molecular Devices Co.).

### 2.6. Statistical Analysis

IBM SPSS Statistics for Windows, Version 24.0 (IBM Corp., Armonk, NY, USA) was used for statistical analyses. An unpaired Student’s *t*-test was used to analyze statistical significance. Data with *p*-values < 0.05 were considered to represent significant differences.

## 3. Results

### 3.1. A. incisum Extract

In the HPLC analysis, the peaks of the 100% methanol extract of *A. incisum* coincided with those of the 75% and 95% ethanol extracts of *A. incisum*, indicating that the eluted components were not different (Figure 1).

### 3.2. Antibacterial Effects and the Sustainability of Antibacterial Activity of A. Incisum on P. gingivalis

**Figure 2:**
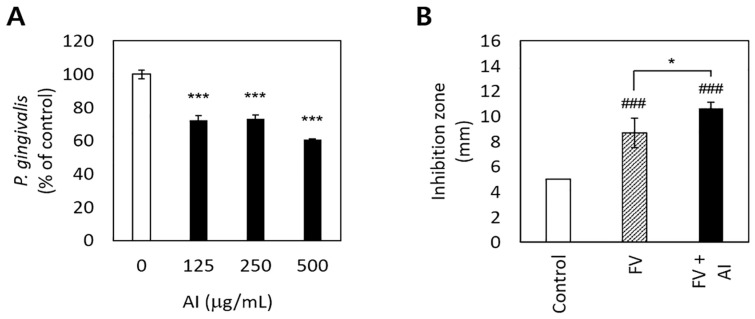
Antibacterial effects of and the sustainability of antibacterial activity by *Asplenium incisum* (AI). (**A**) Inhibitory effects on *Porphyromonas gingivalis* growth according to the concentration of the AI extract. (**B**) Sustained inhibitory effects of AI against *P. gingivalis* on the agar diffusion test after 3 days. *** Indicates a significant difference from the 0 μg/mL AI extract group (*p* < 0.001). ### Indicates a significant difference from the control (film disc per se) group (*p* < 0.001). * Indicates a significant difference between the fluoride varnish (FV) and FV + AI groups (*p* < 0.05).

*A. incisum* significantly decreased the proliferation of *P. gingivalis* (*p* < 0.001) (Figure 2A). In particular, 500 μg/mL of the *A. incisum* extract suppressed the proliferation of *P. gingivalis* by approximately 40%, while 125 μg/mL and 250 μg/mL of the *A. incisum* extract attenuated this effect by approximately 27–28%. The sustainability of antibacterial activity was significantly higher in the FV + AI group than in the FV group after 3 days (*p* < 0.05) (Figure 2B).

### 3.3. Anti-Inflammatory Activity of A. incisum

**Figure 3:**
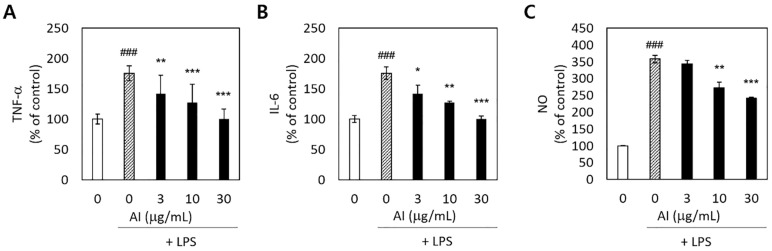
Inhibitory effects of *Asplenium incisum* (AI) on tumor necrosis factor-α (TNF-α) (**A**), interleukin-6 (IL-6) (**B**), and nitric oxide (NO) (**C**) release by lipopolysaccharide (LPS)-stimulated RAW 264.7 cells. ### Indicates a significant difference from the control group without the LPS challenge (*p* < 0.001). * Indicates a significant difference from the 0 μg/mL AI extract group among the LPS-stimulated groups (*p* < 0.05), ** *p* < 0.01, and *** *p* < 0.001.

To determine whether *A. incisum* could decrease proinflammatory reactions in LPS-stimulated RAW 264.7 macrophages, we measured the production of the proinflammatory cytokines TNF-α and IL-6, as well as that of NO. The release of TNF-α, IL-6, and NO was significantly increased by LPS stimulation (*p* < 0.001) (Figure 3). Pretreatment with the *A. incisum* extract significantly decreased the TNF-α and IL-6 levels at all concentrations (*p* < 0.05) (Figure 3A,B). NO production was significantly decreased at *A. incisum* extract concentrations of 10 and 30 μg/mL (*p* < 0.01) (Figure 3C).

### 3.4. Anti-Osteoclastogenic Activity of A. incisum and Its Effect on Cell Viability

**Figure 4:**
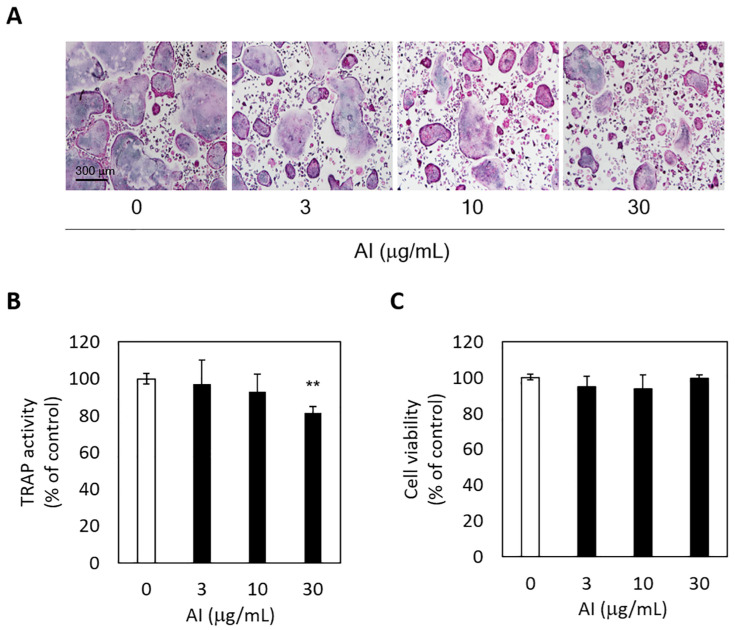
*Asplenium incisum* (AI) suppresses receptor activator of nuclear factor kappa-B ligand (RANKL)-induced osteoclastogenesis. (**A**) To visualize osteoclast differentiation, tartrate-resistant acid phosphatase (TRAP)-positive cell staining was conducted (magnification, ×100). (**B**) TRAP activity was assessed to determine osteoclastogenesis. (**C**) Cell cytotoxicity was analyzed using the Cell Counting Kit-8. ** Indicates a significant difference from the control group (0 μg/mL AI) (*p* < 0.01).

The inhibitory effect of *A. incisum* on RANKL-induced osteoclastogenic differentiation was assessed by observing TRAP-positive multinucleated cells and TRAP activity levels. The *A. incisum* extract significantly attenuated the formation of TRAP-positive multinucleated osteoclasts as its concentration was increased (Figure 4A). TRAP activity was significantly suppressed by the *A. incisum* extract at 30 μg/mL (*p* < 0.01) (Figure 4B). In the cell viability test, the *A. incisum* extract exhibited no cytotoxicity in BMMs (*p* > 0.05) (Figure 4C).

## 4. Discussion

We investigated whether *A. incisum* has inhibitory effects on *P. gingivalis* proliferation, the secretion of NO and inflammatory cytokines (TNF-α and IL-6), and RANKL-induced osteoclastogenesis via TRAP staining and TRAP activity analyses. Our findings revealed that *A. incisum* has antibacterial, anti-inflammatory, and anti-osteoclastogenic activities which can help inhibit periodontal diseases.

*A. incisum* suppressed the growth of *P. gingivalis* and exhibited sustainable antibacterial activity (Figure 2). With respect to the sustainability of antimicrobial activity, FV was used as a carrier of *A. incisum*, and the antibacterial effect lasted for up to 3 days when *A. incisum* was mixed with FV (Figure 2B). In particular, FV may be a useful carrier for antibacterial agents used for improving dental conditions [21]. However, further research is needed to determine the duration of antibacterial activity of FV + AI after 3 days. These results also demonstrate the excellent and sustainable antibacterial activity of *A. incisum* against *P. gingivalis*. In addition, our results highlight the possibility of using FV with antibacterial agents to simultaneously prevent dental caries and periodontal diseases. The active compounds in *A. incisum* are flavonoids such as kaempferol 3-*O*-gentiobioside-4′-*O*-glucoside, kaempferol 3-*O*-glucoside (astragalin), and quercetin 3,4′-diglucoside [22]. Kaempferol and quercetin are often found together in natural products; both of them are the main compounds of *A. incisum* and have antibacterial properties against *Streptococcus mutans* [23]. Since streptococci are some of the early colonizers on the tooth surface and *P. gingivalis* is a late colonizer [24], the inhibition of *S. mutans* leads to the interruption of attachment of *P. gingivalis*. Therefore, *A. incisum* is considered to have antibacterial activities against dental caries and periodontal disease. Although it was difficult to elucidate the antibacterial mechanisms of *A. incisum* with limited data, it is possible that these flavonoids disrupt the bacterial plasma membrane via their phytochemical activities and eventually cause cell death due to cell lysis [25]. Future studies should aim to determine the appropriate concentration of the extract for in vivo use, considering both its antibacterial activity and its cytotoxicity.

The production of the proinflammatory cytokines TNF-α and IL-6 was significantly enhanced by LPS, and *A. incisum* effectively decreased the expression of both TNF-α and IL-6 (Figure 3). Antigen components or other inflammation inducers, such as LPS produced by bacteria, can directly damage the periodontium and cause indirect damage by inducing host immune inflammatory responses [3]. A closer examination of the host immune inflammatory responses revealed that LPS is responsible for the accumulation of inflammatory cells, such as macrophages and dendritic cells [26]. In addition, inflammatory inducers activate intracellular signaling pathways, as well as the secretion of inflammatory mediators, such as TNF-α and ILs [27]. The *A. incisum* extract significantly suppressed the release of TNF-α and IL-6 at increasing concentrations. Previous studies have reported that astragalin, an active compound in *A. incisum*, inhibits the release of TNF-α and IL-6 [28,29].

NO is produced by activated macrophages owing to the activity of inducible NO synthase, and NO acts as a chemical mediator with bactericidal and antitumor activities. However, NO overproduction can induce tissue damage in periodontal tissues, and it has been implicated in the pathogenesis of periodontal diseases [30]. The *A. incisum* extract reduced the LPS-induced release in NO at concentrations of 10 and 30 µg/mL. In other reports, astragalin significantly reduced the secretion of NO in LPS-stimulated RAW 264.7 macrophages [28,29]. In this study, *A. incisum* treatment significantly decreased the expression of TNF-α, IL-6, and NO. Therefore, the inflammatory response induced by LPS in RAW 264.7 cells was clearly attenuated by *A. incisum*.

RANKL-induced osteoclast formation and TRAP activity were also inhibited by *A. incisum*. TRAP staining showed that the number of TRAP-positive cells decreased as the concentration of the *A. incisum* extract increased, and 30 µg/mL of the extract produced a significant decrease in TRAP activity. TRAP has been linked to the physiological function of osteoclasts for decades and is known as a biomarker for bone resorption, which correlates with the number of osteoclasts [30]. The quantified TRAP activity correlated with the number of osteoclasts [31]. Although TRAP is an important cytochemical marker for bone resorption, its clinical application is limited. TRAP activity varies according to age, gender, nutrition, and systemic diseases [32]. Therefore, other osteoclastic parameters such as C-terminal telopeptide of type I collagen and N-terminal telopeptide of type I collagen are used clinically [33]. As shown by the cell cytotoxicity assay, the *A. incisum* extract did not have any significant effect on cell viability after treatment for 72 h at concentrations of up to 30 µg/mL in the culture medium. These results indicate that *A. incisum* has an attenuating effect on osteoclast differentiation without causing any cytotoxicity, and its anti-osteoclastogenetic effect is not induced by cytotoxicity. Nevertheless, our study has a limitation in that the findings of in vitro and animal studies are not always transferable to human physiopathology. Hence, further studies are warranted to investigate the signaling pathways through which *A. incisum* inhibits osteoclastogenesis in periodontal tissues.

These findings indicated that *A. incisum* suppressed the proliferation of *P. gingivalis*, with sustained antibacterial activity for up to 3 days, and it decreased the secretion of proinflammatory cytokines (TNF-α and IL-6), as well as the production of NO. In addition, it suppressed osteoclastogenesis without causing cytotoxicity.

## 5. Conclusions

*A. incisum* was proven to have antibacterial, anti-inflammatory, and anti-osteoclastogenic activities without cytotoxicity in this study. Because *A. incisum,* a natural product, is effective against *P. gingivalis*, it can be used for inhibiting periodontal disease without side-effects, such as those associated with antibiotics. Therefore, *A. incisum* has the potential to be a promising phytotherapeutic candidate for preventing and treating periodontal diseases.

## Data Availability

The data presented in this study are available on request from the corresponding author.

## References

[1] Listgarten M.A. (1986). Pathogenesis of periodontitis. J. Clin. Periodontol..

[2] How K.Y., Song K.P., Chan K.G. (2016). *Porphyromonas gingivalis*: An overview of periodontopathic pathogen below the gum line. Front. Microbiol..

[3] Persson G.R. (2006). What has ageing to do with periodontal health and disease?. Int. Dent. J..

[4] Hienz S.A., Paliwal S., Ivanovski S. (2015). Mechanisms of bone resorption in periodontitis. J. Immunol. Res..

[5] Leitão R.F.C., Ribeiro R.A., Chaves H.V., Rocha F.A.C., Lima V., Brito G.A.C. (2005). Nitric oxide synthase inhibition prevents alveolar bone resorption in experimental periodontitis in rats. J. Periodontol..

[6] Guan X., Zhou Y., Chen H., Wang C., Wang H. (2018). Antibacterial, anti-inflammatory, and anti-osteoclastogenesis roles of allicin in periodontitis. Int. J. Clin. Exp. Med..

[7] Soskolne W.A., Heasman P.A., Stabholz A., Smart G.J., Palmer M., Flashner M., Newman H.N. (1997). Sustained local delivery of chlorhexidine in the treatment of periodontitis: A multi-center study. J. Periodontol..

[8] Tabenski L., Moder D., Cieplik F., Schenke F., Hiller K.A., Buchalla W., Schmalz G., Christgau M. (2017). Antimicrobial photodynamic therapy vs. local minocycline in addition to non-surgical therapy of deep periodontal pockets: A controlled randomized clinical trial. Clin. Oral Investig..

[9] Greenstein G. (1995). Clinical significance of bacterial resistance to tetracyclines in the treatment of periodontal diseases. J. Periodontol..

[10] Teixeira A.H., Freire J.M.O., de Sousa L.H.T., Parente A.T., de Sousa N.A., Arriaga A.M.C., da Silva F.R.L., Melo I.M., da Silva I.I.C., Pereira K.M.A. (2017). *Stemodia maritima* L. extract decreases inflammation, oxidative stress, and alveolar bone loss in an experimental periodontitis rat model. Front. Physiol..

[11] Toker H., Ozan F., Ozer H., Ozdemir H., Eren K., Yeler H. (2008). A morphometric and histopathologic evaluation of the effects of propolis on alveolar bone loss in experimental periodontitis in rats. J. Periodontol..

[12] Yoshinaga Y., Ukai T., Nakatsu S., Kuramoto A., Nagano F., Yoshinaga M., Montenegro J.L., Shiraishi C., Hara Y. (2014). Green tea extract inhibits the onset of periodontal destruction in rat experimental periodontitis. J. Periodontal Res..

[13] Inchingolo F., Tatullo M., Marrelli M., Inchingolo A.M., Picciariello V., Inchingolo A.D., Dipalma G., Vermesan D., Cagiano R. (2010). Clinical trial with bromelain in third molar exodontia. Eur. Rev. Med. Pharmacol. Sci..

[14] Xu Z., Deng M. (2017). Identification and Control of Common Weeds: Vol 2.

[15] Jeong J.G. (2011). A herbalogical study on the plants of Aspleniaceae in Korea. Korea J. Herbol..

[16] Chen C., Peng X., Chen J., Wan C. (2020). Antioxidant, antifungal activities of ethnobotanical *Ficus hirta* Vahl. and analysis of main constituents by HPLC-MS. Biomedicines.

[17] Moon S.H., Ji S.H., Son J.L., Shin S.J., Oh S.H., Kim S., Bae J.M. (2020). Antibacterial, anti-inflammatory, and anti-osteoclastogenic activities of *Colocasia antiquorum* var. *esculenta*: Potential applications in preventing and treating periodontal diseases. Dent. Mater. J..

[18] Lee S.U., Choi Y.H., Kim Y.S., Min Y.K., Rhee M., Kim S.H. (2010). Anti-resorptive saurolactam exhibits in vitro anti-inflammatory activity via ERK-NF-kappaB signaling pathway. Int. Immunopharmacol..

[19] Yeon J.T., Kim K.J., Son Y.J., Park S.J., Kim S.H. (2019). Idelalisib inhibits osteoclast differentiation and pre-osteoclast migration by blocking the PI3Kδ-Akt-c-Fos/NFATc1 signaling cascade. Arch. Pharm. Res..

[20] Moon S.H., Choi S.W., Kim S.H. (2015). In vitro anti-osteoclastogenic activity of p38 inhibitor doramapimod via inhibiting migration of pre-osteoclasts and NFATc1 activity. J. Pharmacol. Sci..

[21] Du M., Cheng N., Tai B., Jiang H., Li J., Bian Z. (2012). Randomized controlled trial on fluoride varnish application for treatment of white spot lesion after fixed orthodontic treatment. Clin. Oral Investig..

[22] Iwashina T., Lopez-Saez J.A., Herrero A., Kitajima J., Matsumoto S. (2000). Flavonol glycosides from *Asplenium foreziense* and its five related taxa and *A. incisum*. Biochem. Syst. Ecol..

[23] Zeng Y., Nikikova A., Abdelsalam H., Li J., Xiao J. (2019). Activity of quercetin and kaemferol against *Streptococcus mutans* biofilm. Arch. Oral Biol..

[24] Perry J.A., Cvitkovitch D.G., Levesque C.M. (2009). Cell death in *Streptococcus mutans* biofilms: A link between CSP and extracellular DNA. FEMS. Microbiol. Lett..

[25] Tagousop C.N., Tamokou J.D., Ekom S.E., Ngnokam D., Voutquenne-Nazabadioko L. (2018). Antimicrobial activities of flavonoid glycosides from *Graptophyllum grandulosum* and their mechanism of antibacterial action. BMC Complement. Altern. Med..

[26] Jia L., Han N., Du J., Guo L., Luo Z., Liu Y. (2019). Pathogenesis of important virulence factors of *Porphyromonas gingivalis* via Toll-like receptors. Front. Cell. Infect. Microbiol..

[27] Chen L., Deng H., Cui H., Fang J., Zuo Z., Deng J. (2017). Inflammatory responses and inflammation-associated diseases in organs. Oncotarget.

[28] Lee H.B., Kim E.K., Park S.J., Bang S.G., Kim T.G., Chung D.W. (2010). Isolation and characterization of nicotiflorin obtained by enzymatic hydrolysis of two precursors in tea seed extract. J. Agric. Food Chem..

[29] Nhiem N.X., Tai B.H., Quang T.H., Kiem P.V., Minh C.V., Nam N.H., Kim J.H., Im L.R., Lee Y.M., Kim Y.H. (2011). A new ursane-type triterpenoid glycoside from *Centella asiatica* leaves modulates the production of nitric oxide and secretion of TNF-alpha in activated RAW 264.7 cells. Bioorg. Med. Chem. Lett..

[30] Uğar-Cankal D., Ozmeric N. (2006). A multifaceted molecule, nitric oxide in oral and periodontal diseases. Clin. Chim. Acta..

[31] Mira-Pascual L., Patlaka C., Desai S., Paulie S., Näreoja T., Lång P., Andersson G. (2020). A novel sandwich ELISA for tartrate-resistant acid phosphatase 5a and 5b protein reveals that both isoforms are secreted by differentiating osteoclasts and correlate to the type I collagen degradation marker CTX-I in vivo and in vitro. Calcif. Tissue Int..

[32] Bernhardt A., Koperski K., Schumacher M., Gelinsky M. (2017). Relevance of osteoclast-specific enzyme activities in cell-based in vitro resorption assays. Eur. Cell. Mater..

[33] Calvo M.S., Eyre D.R., Gundberg C.M. (1996). Molecular basis and clinical application of biological markers of bone turnover. Endocr. Rev..

